# Protein-*O*-fucosylation of coreceptors may be required for Nodal signaling in *Xenopus*

**DOI:** 10.1016/j.mocell.2025.100207

**Published:** 2025-03-03

**Authors:** Yeon-Jin Kim, Seung-Joo Nho, Soo Young Lee, Chang-Yeol Yeo

**Affiliations:** 1 Department of Life Science, College of Natural Sciences, Ewha Womans University, Seoul, Republic of Korea; 2 ICM, Building 102 4th Floor, 50 Yonsei-ro, Seodaemun-gu, Seoul, Republic of Korea; 3 Multitasking Macrophage Research Center, Ewha Womans University, Seoul, Republic of Korea

**Keywords:** Body axis formation, Cripto, Embryogenesis, Smad2, Xnr1

## Abstract

Nodal-related ligands of TGF-β family play pivotal roles for mesoderm induction and body axis formation during vertebrate early embryogenesis. Nodal ligands are distinct from most other TGF-β ligands family as they require EGF-CFC factors as coreceptors for signaling, in addition to their cognate type I and type II TGF-β receptors. In amphibian *Xenopus laevis* embryos, 5 *Nodal-related* genes (*Xnr1/2/4/5/6*) and 2 *EGF-CFC* genes (*XCR1*, *XCR3*) play roles in mesoderm induction and the accumulation of phosphorylated Smad2, while in mammalian embryos, 1 *Nodal* gene and 1 *EGF-CFC* gene (*Cripto*) play roles during mesoderm induction. Mammalian EGF-CFC factors are reported to be *O*-fucosylated at a conserved threonine residue of the EGF-like motif by protein-*O*-fucosyltransferase 1 (Pofut1), but this *O*-fucose modification is shown to be dispensable for Nodal signaling in mammalian embryos. In this study, we investigated the developmental roles of *Xenopus laevis Pofut1* (*XPofut1*) and its potential function in Nodal signaling. We found that morpholino antisense-mediated knockdown of *XPofut1* causes reduction of Smad2 phosphorylation in late blastula and axial truncation in neurula. We also found that the *O*-fucosyltransferase activity of XPofut1 is important in the marginal zone, but not in the vegetal pole region, of blastula. Interestingly, *XPofut1* is necessary for Smad2 phosphorylation induced by Xnr1 or Xnr2, but not by Xnr5 or Xnr6. Among the Nodal signaling components, only EGF-CFC factors are known to be modified by Pofut1. Therefore, based on our current observation, we propose that XPofut1 regulates signaling of a subset of nodal ligands in pregastrulation embryos possibly through modulating the function of EGF-CFC factors.

## INTRODUCTION

Nodal ligands are members of the TGF-β family and they play pivotal roles during vertebrate embryogenesis (Hill, 2018, Hill, 2022, Jones and Mullins, 2022). During early embryogenesis, *Nodal* and *nodal-related* genes are essential for the specification of dorsal-ventral body axis and the induction of mesoderm. In amphibian *Xenopus laevis*, 6 *Xenopus nodal-related* genes (*Xnr1∼6*) have been identified and, besides *Xnr3*, they all share mesoderm-inducing activity (Carron and Shi, 2016, Luxardi et al., 2010, Skirkanich et al., 2011, Tadjuidje et al., 2016, Takahashi et al., 2000). *Xnr5* and *Xnr6* are expressed before the mid blastula transition (MBT) and they are required, after the MBT, for the expression of *Xnr1*, *Xnr2,* and *Xnr4* and for the accumulation of phosphorylated Smad2, a hallmark of Nodal signaling.

Nodal ligands bind to their cognate type I (Acvr1b and Acvr1c) and type II (Acvr2a and Acvr2b) TGF-β receptors leading to the subsequent phosphorylation of intracellular Smad2 and Smad3 (Hill, 2018, Hill, 2022). Phosphorylated Smad2/3 interact with Smad4 in the cytoplasm, enter the nucleus, form transcriptional complexes with DNA-binding proteins, and induce the expression of target genes.

Nodal ligands, unlike other TGF-β molecules, also require EGF-CFC factors as coreceptors (Calvanese et al., 2015, Dorey and Hill, 2006, Minchiotti et al., 2001, Onuma et al., 2006, Yabe et al., 2003). Mammals have 2 *EGF-CFC* genes (*Cripto*, *Cryptic*) and *Xenopus laevis* has 3 *EGF-CFC* genes (*XCR1*, *XCR2*, and *XCR3*) with distinct spatiotemporal expression patterns. EGF-CFC factors contain 2 conserved domains, an EGF-like domain, and a CFC domain, and they are tethered to the plasma membrane by a GPI linkage. There are 2 different hypotheses of how EGF-CFC factors function in Nodal signaling. At first, EGF-CFC factors, acting as coreceptors, were shown to promote the binding of Nodal ligands to the receptor (Calvanese et al., 2015, Minchiotti et al., 2001, Watanabe et al., 2007, Yeo et al., 1999). In an alternative model, EGF-CFC factors were shown to be necessary for the processing and internalization of Nodal ligands into the endosome, where Nodal signaling is thought to predominantly occur (Blanchet et al., 2008a, Blanchet et al., 2008b).

Human and mouse Cripto proteins have been shown to undergo *O*-linked fucose modification. *O*-fucosylation is mediated by protein-*O*-fucosyltransferase 1 and 2 (Pofut1/2), and occurs at conserved Ser/Thr residues in EGF-like repeats (C_2_-XXXX**S/T**-C_3_) of a handful of proteins, including plasminogen activators, blood clotting factors, and Notch proteins. In *Drosophila*, Pofut1 alters the ligand-binding specificity of Notch (Pandey et al., 2019). *O*-fucosylation of Cripto occurs at Thr88 residue in the ligand-binding EGF-like domain, and substitution of Thr88 to Ala renders Cripto nonfunctional for Nodal signaling (Schiffer et al., 2001). However, a detailed study suggests that *Pofut1* is dispensable for Nodal signaling in mammals and that the Thr88 residue itself but not the *O*-fucose is important for Nodal signaling (Shi et al., 2007).

In this study, we investigated the developmental roles of *Xenopus laevis Pofut1* (*XPofut1*) and its potential roles in Xnr signaling. *XPofut1* knockdown with an antisense morpholino oligonucleotide (MO) caused aberrant patterns of Smad2 phosphorylation and defects in anterior-posterior patterning. Of interest, we found that *XPofut1* differentially regulates Xnr1/2 vs Xnr5/6 signaling and the accumulation of phosphorylated Smad2 in different regions of the embryo. These results suggest that *XPofut1* is important for signaling of at least a subset of Xnr ligands during early embryogenesis.

## MATERIALS AND METHODS

### Plasmids

The open-reading frame of *XPofut1* was subcloned into pCS2+ vector. For Sec-XPofut1 (*Xenopus laevis* Pofut1) and Sec-hPOFUT1 (human POFUT1), the signal peptide sequence of XPofut1 or hPOFUT1 was replaced with the human IgΚ signal peptide sequence. For glycosyltransferase-defective hPOFUT1, Arg127 residue was substituted to Ala.

### Morpholino Oligonucleotides

Standard MO, random MO (negative controls), and gene-specific MOs used in this study were purchased from Gene Tools, LLC. The following gene-specific MOs were used:

**Table:** 

Name	Sequence (sequence complementary to the start codon is bold)
*XPofut1* MO	5′-AAG CCA AAC ACC GCG CTC **CAT** TCC C-3′
*XCR1* MO1	5′-AAA CTG CAT TGT TTT CTG CAA AGG C-3′
*XCR1* MO2	5′-ATT TAA TGT GTC CTC AGC AAA AGC C-3′
*XCR3* MO1	5′-CAT GGC ACA GTC CTG CTC CAA CTA A-3′
*XCR3* MO2	5′-CCA TAC CAT GGC ACA GTC CTG CTC C-3′

### *Xenopus laevis* Embryo Manipulation and Microinjection

Fertilized embryos were dejellied in 3% cysteine/0.1× MMR, microinjected, and cultured at 0.33× MMR. Animal caps were dissected at stage 8.5 and cultured in 0.7× MMR. Staging of embryos was according to Nieuwkoop and Faber (Zahn et al., 2022). Synthetic mRNAs were transcribed with SP6 mMessage mMachine kit (Ambion). For microinjection into 2-cell- or 4-cell-stage embryos, MO and mRNA were injected into each blastomere.

### Whole-Mount *In Situ* Hybridization

*In situ* hybridization was performed as previously described (Harland, 1991). Digoxigenin (DIG)-labeled RNA probe, containing the antisense sequence of the entire coding region of *XPofut1*, was synthesized using DIG-UTP (Roche Life Science) and MEGAscript SP6 kit (Ambion). Embryos of selected stages were fixed in MEMFA, dehydrated in methanol, and rehydrated in PTw (1× PBS, 0.1% Tween-20). Embryos were treated with 10 μg/mL of proteinase K (Roche Life Science) for 5 minutes followed by prehybridization. Hybridization was carried out at 62°C overnight. The DIG-labeled probes were visualized using anti–DIG-AP-Fab fragment and BM purple substrate (Roche Life Science).

### Quantitative RT-PCR

Embryos and explants of indicated stages were treated with RNAlater (Ambion) overnight at 4°C. Total RNA was isolated using RNeasy Mini Kit (Qiagen) and reverse-transcribed using Superscript III First-strand Synthesis System (Invitrogen). Real-time PCR was performed in Rotor-gene 6 instrument (Corbett Research) using QuantiTect SYBR Green PCR Kit (Qiagen).

A representative result of 3 separate experiments is shown. The following primers were used:

**Table:** 

Gene	Direction	Sequence
*Eomesodermin* (*Eomes*)	Forward	5′-GCA GAG GTT CTA CCT ATC-3′
	Reverse	5′-CAT TGC TTG AGG TGC TAG G-3′
*Goosecoid* (*Gsc*)	Forward	5′-ACA ACT GGA AGC ACT GGA-3′
	Reverse	5′-TCT TAT TCC AGA GGA ACC-3′
*EF1α*	Forward	5′-CCT GAA CCA CCC AGG CCA GAT TGG TG-3′
	Reverse	5′-GAG GGT AGT CAG AGA AGC TCT CCA CG-3′
*VegT*	Forward	5′-CAA GTA AAT GTG AGA AAC CGT G-3′
	Reverse	5′-CAA ATACAC ACA CAT TTC CCG-3′
*Xbra*	Forward	5′-GGA TCG TTA TCA CCT CTG-3′
	Reverse	5′-GTG TAG TCT GTA GCA-3′
*Xnr1*	Forward	5′-AAC CAT CAC TTA TCA ATA GG-3′
	Reverse	5′-TGT AGG CCA GTA AAA TCA TTA AC-3′
*XPofut1*	Forward	5′-GAT GCC AGG CGG GTG TCT TGT TT-3′
	Reverse	5′-AGG CCT GAT TTC ATG GAG TCT TTA-3′
*XVent1*	Forward	5′-TTC CCT TCA GCA TGG TTC AAC-3′
	Reverse	5′-GCA TCT CCT TGG CAT ATT TGG-3′

### Luciferase Assay

Firefly luciferase reporter A3-Luc (Yeo et al., 1999), containing 3 tandem repeats of the activin-response element, and control reporter pRL-CMV (50 pg each/embryo) were injected to 2-cell-stage embryos. Luciferase assays were performed using Dual Luciferase Reporter Assay System (Promega). Firefly luciferase activities were normalized to the corresponding *Renilla* luciferase activities. A representative result of 3 separate experiments is shown.

### Immunoblotting

Embryos or animal caps at indicated stages were lyzed in a volume of 20 μL/embryo or 2 μL/animal cap with an ice-cold lysis buffer [50 mM Tris HCl (pH 8.0), 150 mM NaCl, 1% NP40, 0.5% sodium deoxycholate, 2 mM EDTA, 10% glycerol, 0.1% SDS, 2 mM β-glycerophosphate, 2 mM imidazole, 10 mM sodium fluoride, 1.15 mM sodium molybdate, 1 mM sodium orthovanadate, 2 mM sodium pyrophosphate, 4 mM sodium tartrate dehydrate, 10 nM calyculin A, 30 nM okadaic acid, 1× Cømplete protease inhibitor cocktail-EDTA (Roche), 1 mM NEM, and 1 mM PMSF]. After centrifugation, supernatants were subjected to SDS-PAGE, and proteins were transferred to nitrocellulose membrane and visualized using appropriate antibodies and ECL reagent. The following antibodies were used: antiphospho-Smad2 (Ser465/467) (Cat. # 3101, Cell Signaling Technology), anti-Smad2/3 (Cat. # 610842, BD Bioscience), and antiactin (Cat. # A4700, Sigma-Aldrich). Total Smad2 and actin were used as loading controls. Each experiment is repeated at least 3 times and the representative results are shown.

## RESULTS

### Expression Patterns of *XPofut1*

*Xenopus laevis pofut1 L* transcript (GenBank accession # NM_001088891) encodes a 380-amino acid long polypeptide (GenBank accession # NP_001082360), and its amino acid sequence is 71.82% (85%) and 73% (85%) identical to human and mouse Pofut1, respectively (numbers in parentheses are similarities) (Supplementary Fig. 1). We named this protein *Xenopus laevis* protein-*O*-fucosyltransferase 1 (XPofut1). XPofut1, like its mammalian homologs, contains an N-terminal signal peptide, a glycosyltransferase domain, and a C-terminal KDEL-like ER retention sequence.

**Fig. 1:**
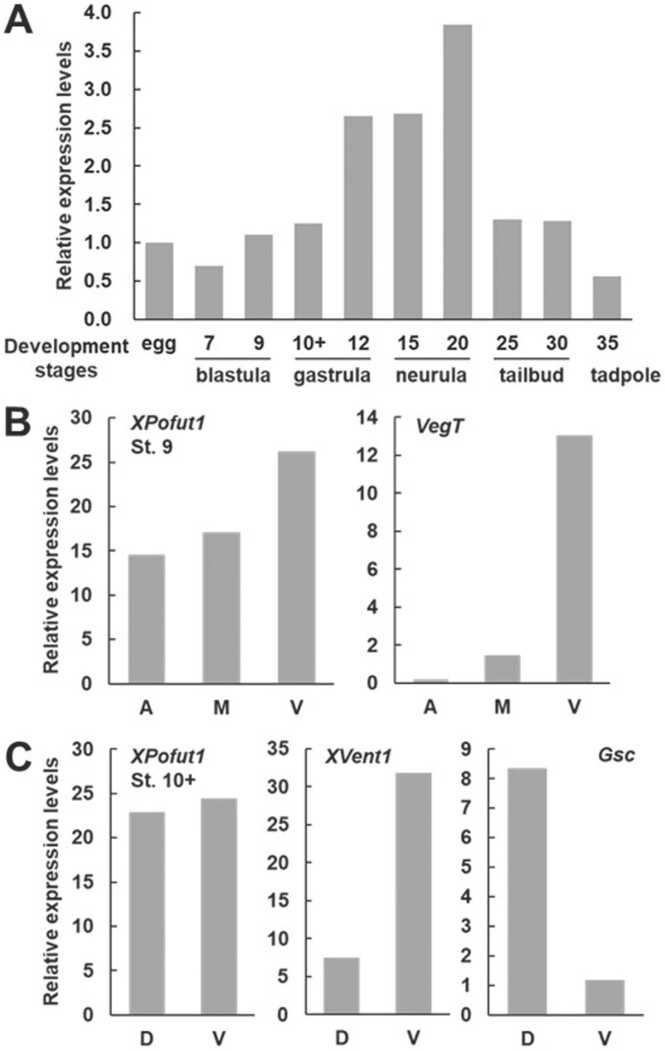
Expression patterns of *XPofut1*. Total RNAs from embryos or regions of embryos at indicated stages were isolated, the transcript levels of *XPofut1* were analyzed by RT-PCR and normalized with those of *EF1α*. (A) Comparison of the levels of *XPofut1* transcripts in unfertilized egg, blastula (stages 7 and 9), gastrula (stages 10+ and 12), neurula (stages 15 and 20), tailbud (stages 25 and 30), and tadpole (stage 35). (B) Stage 9 embryos were divided into the animal (A), marginal (M), and vegetal (V) regions. The levels of *VegT* (a ventral marker) transcripts are compared to confirm the accuracy of dissection. (C) Stage 10 embryos were divided into the dorsal (D) and vegetal (V) halves. The levels of *Gsc* (a dorsal marker) and *XVent1* (a ventral marker) transcripts are compared to confirm the accuracy of dissection.

**Fig. 2:**
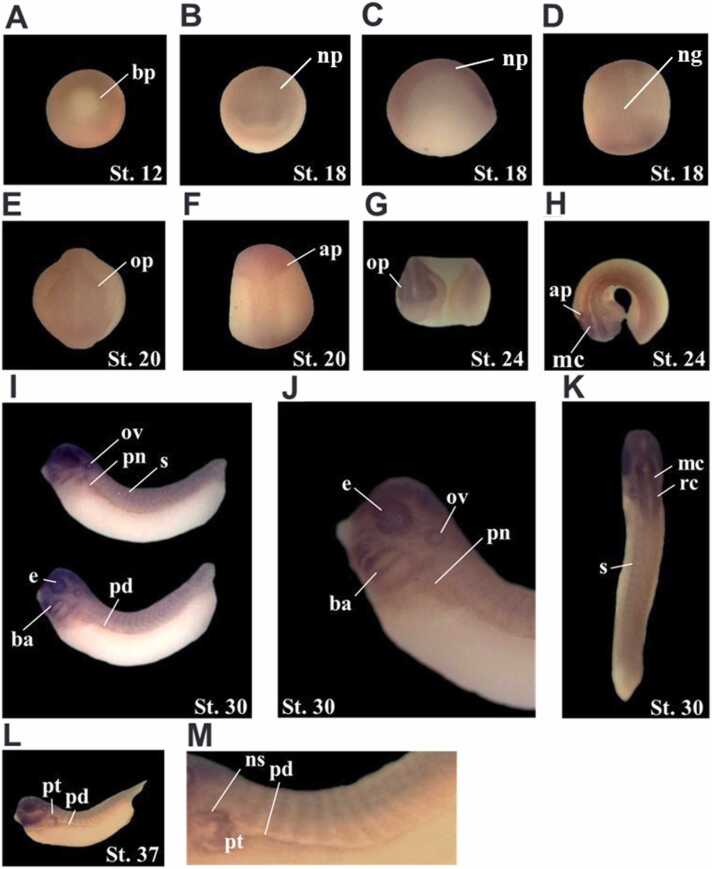
Spatial expression patterns of *XPofut1*. Whole-mount *in situ* hybridization was performed with embryos of indicated stages. Photographs of representative embryos are shown. (A) Stage 12, vegetal view, dorsal side is toward the top. (B-D) Stage 18, anterior view, dorsal side is toward the top (B), lateral view, dorsal side is toward the top (C), and dorsal view, anterior side is toward the top (D). (E and F) Stage 20, anterior view, dorsal side is toward the top (E) and dorsal view, anterior side is toward the top (F). (G and H) Stage 24, lateral view (G), and dorsal view (H). (I-K) Stage 30, lateral view (I), higher magnification of anterior region (J), and dorsal view (K). (L and M) Stage 37, lateral view (L) and higher magnification of dorsal region (M). ap, auditory placode; ba, branchial arch; bp, blastopore; e, eye; mc, mesencephalon, ng, neural groove; np, neural plate; ns, nephrostome; op, optic placode; ov, optic vesicle; pd, pronephric duct; pn, pronephros; pt, pronephric tubule; rc, rhombencephalon; s, somite.

We first analyzed the temporal and spatial expression patterns of *XPofut1*. *XPofut1* transcripts are present in unfertilized eggs, and the levels of transcript increase gradually from the late blastula stage (stage 9), peak at the neurula stage (stage 20), and gradually decrease until the tadpole stage (stage 35) (Fig. 1A). In late blastula (stage 9), *XPofut1* transcripts are present throughout the animal, marginal, and vegetal regions with slightly higher levels in the vegetal region (Fig. 1B). In early gastrula (stage 10+), *XPofut1* transcripts are distributed evenly across the dorsal-ventral axis (Fig. 1C). According to the results of *in situ* hybridization analyses, *XPofut1* expression becomes restricted to specific regions of embryos as development progresses (Fig. 2). *XPofut1* transcripts are localized around the blastopore in late gastrula (stage 12) and to the anterior neural plate in neurula (stages 18 and 20). At the tailbud (stages 24-30) and tadpole (stage 37) stages, *XPofut1* expression is significantly higher in the anterior-dorsal region than the posterior-ventral region and becomes restricted to the brain, eye, branchial arches, pronephros, and somites.

### *XPofut1* Is Important for Axial Patterning

The developmental role of *XPofut1* is assessed using antisense MO-mediated knockdown. *XPofut1* MO blocked *in vitro* translation of *XPofut1* mRNA, which contains the target sequence of MO, but not the translation of mutant *XPofut1* mRNA containing alternative codons in the MO target sequence (Supplementary Fig. 2). A negative control MO (*XPofut1-*sense MO), containing the complementary sequence of *XPofut1* MO, had no effect on the translation of wild-type *XPofut1* mRNA (Supplementary Fig. 2).

**Fig. 3:**
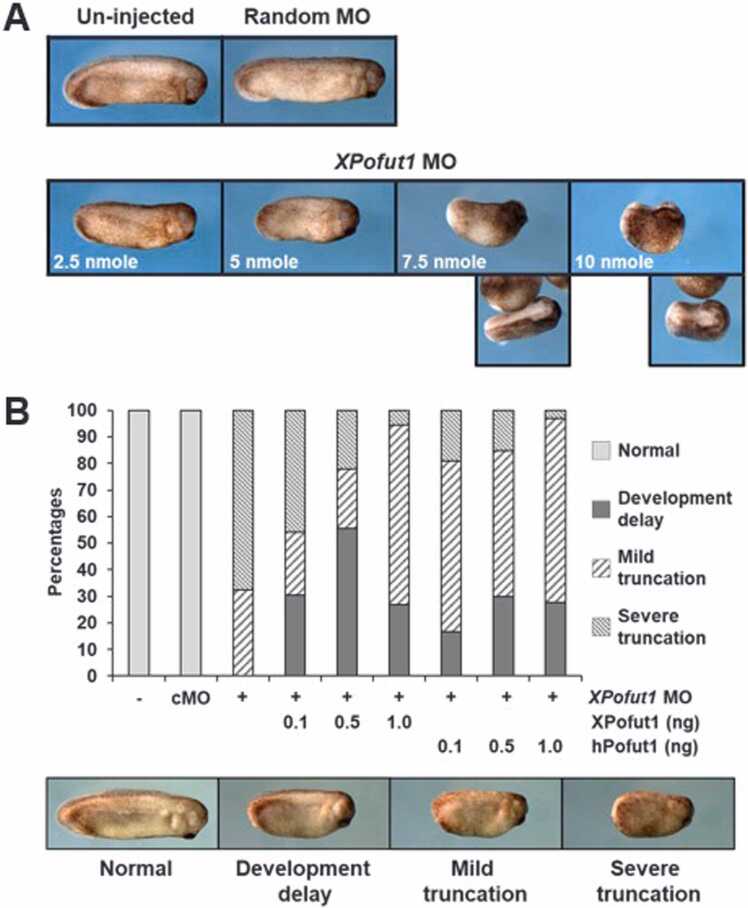
*XPofut1* knockdown causes axial truncation. (A) Indicated amounts of *XPofut1* (2.5-10 nmole/embryo) or control random MO (10 nmole/embryo) were injected into the marginal regions at 2-cell stage and the embryos were cultured until stage 27. Photographs of representative embryos are shown. (B) Indicated combinations of *XPofut1* or control random MO (cMO) (5 nmole/embryo), Sec-hPOFUT1 or Sec-XPofut1 mRNA (ng/embryo) were coinjected into the marginal regions at 2-cell stage. The embryos were cultured until stage 27 and scored for phenotypes. Photographs of representative embryos for each category of phenotype are shown, and percentages of embryos to each category are tabulated.

When microinjected into both blastomeres of 2-cell-stage embryos, *XPofut1* MO did not cause any discernible defects until the onset of gastrulation (data not shown). However, *XPofut1* MO caused apparent defects as development progressed and, at the tadpole and tailbud stages, *XPofut1* morphants showed anterior and posterior truncation of the body axis in a dose-dependent manner (Fig. 3A). A control random MO did not cause any discernible defects in tadpoles and tailbud embryos. Defects in *XPofut1* morphants could be partially rescued by coinjection of recombinant *XPofut1* (Sec-XPofut1) or human *POFUT1* (Sec-hPOFUT1) mRNA encoding XPofut1/hPOFUT1 proteins with an *XPofut1* MO nontargeted heterologous signal peptide (Fig. 3B). These results suggest that *XPofut1* is involved in the axial patterning.

The axial truncation in *XPofut1* morphants was resembling those of *XCR1* and *XCR3* morphants although the defects in *XPofut1* morphants were less severe (Supplementary Fig. 3). Knockdown of both *XCR1* and *XCR3* caused defects at lower doses than knockdown of both *XCR1* or *XCR3* alone as previously reported (Dorey and Hill, 2006).

### *XPofut1* Is Important for Xnr1 Signal Transduction

**Fig. 4:**
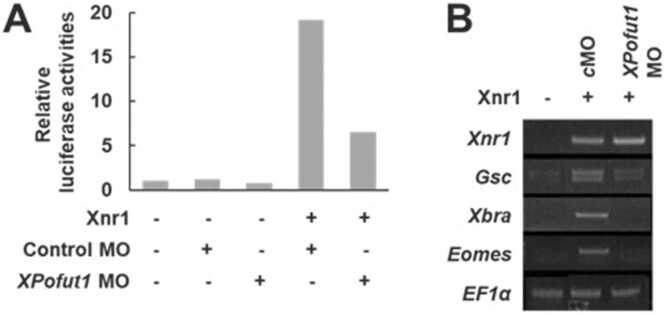
*XPofut1* is involved in Xnr1 signal transduction. (A) A3-Luc reporter plasmid (50 pg/embryo) was injected into animal regions at 2-cell stage with indicated combinations of control random or *XPofut1* MO (5 nmole/embryo) and Xnr1 mRNA (125 pg/embryo). Animal caps were explanted at stage 8.5, cultured until stage 9.5, and harvested to analyze luciferase activities. (B) Indicated combinations of control random (cMO) or *XPofut1* MO (5 nmole/embryo) and Xnr1 mRNA (125 pg/embryo) were injected into animal regions of 2-cell-stage embryos. Animal caps were explanted at stage 8.5, cultured until stage 9.5, and harvested for RT-PCR analysis. *EF1α* is used as a loading control.

To determine whether *XPofut1* is involved in Nodal signaling, we first examine the effects of *XPofut1* knockdown on the expression of a Nodal-responsive luciferase reporter A3-Luc. A3-Luc contains 3 tandem repeats of the activin-response element, and activin-response element contains binding sites for the FoxH1/pSmad2/Smad4 complex, a transcriptional complex formed in *Xenopus* embryos in response to nodal/activin stimuli (Yeo et al., 1999). *XPofut1* MO reduced the response of A3-Luc to Xnr1 (Fig. 4A). *XPofut1* MO also inhibited the expression of Xnr1 target genes *Goosecoid* (*Gsc*), *Brachyury* (*Xbra*), and *Eomesodermin* (*Eomes*) in ectodermal explant (animal cap) assay (Fig. 4B). These results suggest that *XPofut1* is important for Xnr1 signaling.

### *XPofut1* Is Important for the Accumulation of Phosphorylated Smad2 During Embryogenesis

**Fig. 5:**
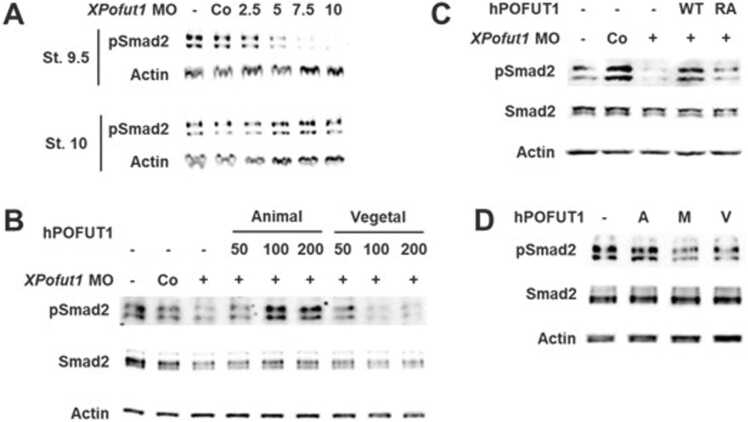
*XPofut1* is important for Smad2 phosphorylation in early embryos. (A) Two-cell-stage embryos were injected into marginal regions with random MO (Co, 10 nmole/embryo) or indicated amounts of *XPofut1* MO (nmole/embryo), and cultured until stage 9.5 or 10. The levels of phosphorylated Smad2 (pSmad2) are compared by immunoblotting. Actin is used as loading control. (B-D) Microinjected embryos were cultured until stage 9.5 and harvested to analyze the levels of phosphorylated Smad2 (pSmad2) by immunoblotting. Levels of total Smad2 (Smad2) are also compared and actin is used as a loading control. (B) Random control (Co) or *XPofut1* MO (5 nmole/embryo) was injected into marginal regions at 2-cell stage, and then indicated amounts (pg/embryo) of *hPOFUT1* mRNA were injected into either 4 animal blastomeres (Animal) or 4 vegetal blastomeres (Vegetal) at 8-cell stage. (C) Random control or *XPofut1* MO (5 nmole/embryo) was injected into the marginal region at 2-cell stage, and then mRNA encoding wild-type (WT) or glycosyltransferase-defective mutant form (RA) of hPOFUT1 (100 pg/embryo) was injected into 4 animal blastomeres at 8-cell stage. (D) hPOFUT1 mRNA (100 pg/embryo) was injected into 4 animal blastomeres at 8-cell stage (A), into marginal regions at 2-cell stage (M), or into 4 vegetal blastomeres at 8-cell stage (V).

Activation of Nodal signaling in embryos leads to the accumulation of Smad2 that is phosphorylated (pSmad2) at the C-terminal SSXS motif (Hill, 2018). To determine whether *XPofut1* is involved in Nodal signaling during development, we examined the effects of *XPofut1* MO on pSmad2 accumulation in embryos. *XPofut1* MO, but not a control random MO, reduced pSmad2 accumulation in stage 9.5 embryos in a dose-dependent manner (Fig. 5A). However, in stage 10 *XPofut1* morphants, levels of pSmad2 had recovered to levels comparable to those of sibling wild-type embryos (Fig. 5A). In pregastrula-stage embryos, Xnr signaling, measured by Smad2 phosphorylation, is activated first in the dorsal side, and gradually propagates to the marginal sides and to the ventral sides as development progresses (Carron and Shi, 2016, Faure et al., 2000, Lee et al., 2001). As gastrulation progresses in stage 10 embryos, negative feedback regulation of Xnr signaling, through the expression of Xnr antagonists, reduces Smad2 phosphorylation from the dorsal side first and this reduction in Smad2 phosphorylation also propagates to the marginal and ventral sides as development progresses. Therefore, the recovery of pSmad2 accumulation in stage 10 *XPofut1* morphants could result from incomplete *XPofut1* knockdown or the absence of negative feedback regulation of Xnr signaling. Nevertheless, the delayed accumulation of pSmad2 was enough to cause axial truncation in *XPofut1* morphants in tailbud- and tadpole-stage embryos (Fig. 3A).

We examined if the reduction of pSmad2 accumulation in *XPofut1* morphants can be relieved with coinjection of MO nontargeted recombinant *Xenopus Pofut1* (Sec-*XPofut1*) or human *POFUT1* (*hPOFUT1*) mRNA. When microinjected to the marginal region of 2-cell-stage *XPofut1* morphants, XPofut1 or hPOFUT1 did not reverse the effects of *XPofut1* MO on Smad2 phosphorylation (data not shown). mRNAs microinjected to the marginal region of the 2-cell-stage embryos can diffuse throughout the embryo. We examined the effects of restricting the distribution of Pofut1 to the animal or vegetal half of an embryo by microinjection of *hPOFUT1* mRNA only in the animal blastomere or vegetal blastomere of 8-cell-stage embryos. mRNA microinjected to animal blastomere of 8-cell-stage embryos would be restricted to animal cap region and marginal zone in stages 8 to 10 embryos (blastula to early gastrula), whereas mRNA microinjected to vegetal blastomere would be distributed to marginal zone and vegetal pole regions in stages 8 to 10 embryos. Microinjection of *hPOFUT1* mRNA to the animal half of *XPofut1* morphants increased Smad2 phosphorylation in a dose-dependent manner, whereas microinjection of *hPOFUT1* mRNA to the vegetal half of *XPofut1* morphants did not cause discernible change on Smad2 phosphorylation (Fig. 5B). Sec-*XPofut1* mRNA had similar effects (data not shown). Since Smad2 phosphorylation occurs only in the marginal zone (presumptive mesoderm) and the vegetal pole region (presumptive endoderm) but not in the animal cap region (presumptive ectoderm, Xnr ligands not present) in stages 8 to 10 embryos, recovery of Smad2 phosphorylation by expression of hPOFUT1 in the animal half of *XPofut1* morphants is likely due to the recovery of Smad2 phosphorylation in the marginal zone. These results suggest that, for the accumulation of Smad2 phosphorylation in blastula/early gastrula, XPofut1 activity is required only in the marginal zone but not in the vegetal pole region even when *XPofut1* transcripts are distributed throughout the embryo.

Next, we investigated whether the glycosyltransferase activity of Pofut1 is necessary for the reversion of pSmad2 reduction caused by *XPofut1* knockdown. When overexpressed only in the animal half of an embryo, hPOFUT1 RA mutant, with an Arg-to-Ala substitution of Arg127 residue important for the glycosyltransferase activity, was unable to increase Smad2 phosphorylation in *XPofut1* morphants (Fig. 5C). We also examined what effects does Pofut1 overexpression in different regions of the embryo have on Smad2 phosphorylation. When microinjected to the animal half, *hPOFUT1* mRNA did not cause any discernible change in Smad2 phosphorylation at stage 9.5 (Fig. 5D). However, microinjection of *hPOFUT1* mRNA to the marginal region at 2-cell stage (mRNA is distributed throughout the embryo) or to vegetal half slightly, but reproducibly, decreased Smad2 phosphorylation at stage 9.5 (Fig. 5D). These results suggest that the glycosyltransferase activity of XPofut1 is important for the accumulation of pSmad2 in the marginal zone, while XPofut1 is unnecessary or inhibitory to the accumulation of pSmad2 in the vegetal pole region at least prior to the onset of gastrulation.

We also examined if activation of other signaling pathways, that are important for axial patterning in *Xenopus* embryos, can reverse the effect of *XPofut1* MO on Xnr1-induced Smad2 phosphorylation. FGF and Wnt signaling pathways coordinate with Nodal signaling pathway in the control of embryonic body axis formation, germ layer specification, and gastrulation movement (Jones and Mullins, 2022). We also examined the effects of Notch signaling pathway activation in *XPofut1* morphants as Pofut1 has been shown to modulate Notch signaling in mammalian embryos and as Notch signaling pathway plays important roles in early embryogenesis (Pandey et al., 2019). As expected, src-hALK4(TD), a constitutively active recombinant form of Acvr1b (type I receptor for Nodal ligands), significantly increased Smad2 phosphorylation in *XPofut1* morphants (Supplementary Fig. 4). However, neither the activation of FGF signaling pathway with overexpression of constitutively active Ras (H-RasV12), Wnt signaling pathway with overexpression of β-catenin, nor Notch signaling pathway with overexpression of Notch intracellular domain caused any discernible change in the reduction of Smad2 phosphorylation caused by *XPofut1* knockdown (Supplementary Fig. 4).

### *XPofut1* Is Necessary for Xnr1/2 Signaling but Not for Xnr5/6 Signaling

**Fig. 6:**
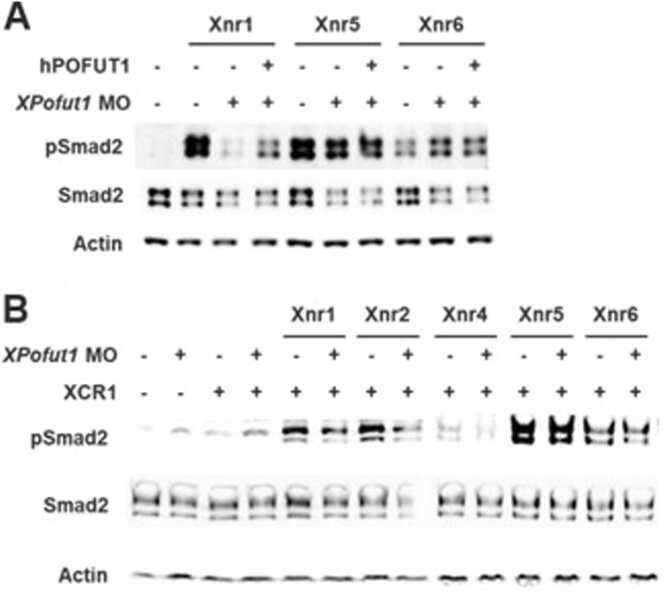
*XPofut1* regulates Xnr1 and Xnr5/6 signaling differently. Embryos were injected with indicated combinations of MO and mRNA. Animal caps were explanted at stage 8.5, cultured until stage 9.5, and harvested to analyze the levels of phosphorylated Smad2 (pSmad2) by immunoblotting. Levels of total Smad2 (Smad2) are also compared and actin is used as a loading control. (A) Embryos were injected into marginal regions with indicated combinations of *XPofut1* MO (5 nmole/embryo) and mRNAs encoding Xnr1 (125 pg/embryo), Xnr5 (40 pg/embryo), or Xnr6 (300 pg/embryo) at 2-cell stage, and then injected into 4 animal blastomeres with hPOFUT1 mRNA (100 pg/embryo) at 8-cell stage. (B) Embryos were injected into marginal regions with indicated combinations of *XPofut1* MO (5 nmole/embryo) and mRNAs encoding Xnr1 (125 pg/embryo), Xnr2 (20 pg/embryo), Xnr4 (100 pg/embryo), Xnr5 (40 pg/embryo), Xnr6 (300 pg/embryo), or XCR1 (250 pg/embryo) at 2-cell stage.

*Xnr5/6* transcripts are accumulated and restricted to the vegetal region of pre-MBT embryos, and *Xnr5/6* induce the expression of *Xnr1/2* at the onset of MBT. *Xnr1/2* transcripts become restricted to the marginal zone as development progresses (Luxardi et al., 2010, Takahashi et al., 2000). Since overexpressing Pofut1 in animal vs vegetal half of an embryo resulted in different outcomes, we examined the effects of XPofut1 knockdown on the signal transduction of Xnr1, Xnr2, Xnr5, and Xnr6. In animal cap assays, *XPofut1* MO blocked Xnr1-induced Smad2 phosphorylation and coinjection of *hPOFUT1* mRNA reversed the effect of *XPofut1* MO on Smad2 phosphorylation (Fig. 6A). However, neither *XPofut1* MO nor coinjection of *hPOFUT1* mRNA caused any discernible change in Xnr5 or Xnr6-induced Smad2 phosphorylation (Fig. 6A). Next, we examined the effects of *XPofut1* MO on Xnr-induced Smad2 phosphorylation in the presence of XCR1. *XPofut1* MO reduced Xnr1 or Xnr2-induced Smad2 phosphorylation when XCR1 is coexpressed, whereas *XPofut1* MO had no effect on Xnr5 or Xnr6-induced Smad2 phosphorylation (Fig. 6B). These results suggest that *XPofut1* is important for Xnr1 and Xnr2 signal transduction, whereas it is not required for Xnr5 or Xnr6 signal transduction.

We found that the glycosyltransferase activity of XPofut1 is important for the accumulation of pSmad2 in embryo (Fig. 5C). Among the components of Nodal signal transduction pathway, only EGF-CFC factors are shown to be *O*-fucosylated so far (Schiffer et al., 2001, Shi et al., 2007). Of 3 *EGF-CFC* factors in *Xenopus laevis*, *XCR1* and *XCR3* are shown to be expressed and functional in pregastrulation embryos (Dorey and Hill, 2006). We examined if XPofut1 interacts with XCR proteins. XPofut1 interacted with all 3 XCR proteins, with significantly weaker interaction for XCR2 than XCR1 or XCR3 (Supplementary Fig. 5).

## DISCUSSION

We propose that *XPofut1* is important for signaling by a subset Xnr ligands during early embryogenesis of *Xenopus laevis*, based on the fact that *XPofut1* knockdown causes axial truncation, the reduction of Smad2 phosphorylation, and the decrease in the expression of Nodal target genes and a Nodal-responsive reporter. Interestingly, we found that *XPofut1* is required for the accumulation of phosphorylated Smad2 (pSmad2) in the marginal zone in pregastrulation embryos, whereas *XPofut1* is unnecessary or inhibitory for the accumulation of pSmad2 in the vegetal pole region.

### Roles of *XPofut1* During Embryogenesis

Our results indicate that *XPofut1* is important for the accumulation of pSmad2 in pregastrulation embryos and axial patterning. During early development in *Xenopus laevis*, Xnr signaling is necessary for the accumulation of pSmad2 (Luxardi et al., 2010, Skirkanich et al., 2011, Tadjuidje et al., 2016, Takahashi et al., 2000). These results suggest that *XPofut1* plays important roles in Xnr signal transduction.

**Fig. 7:**
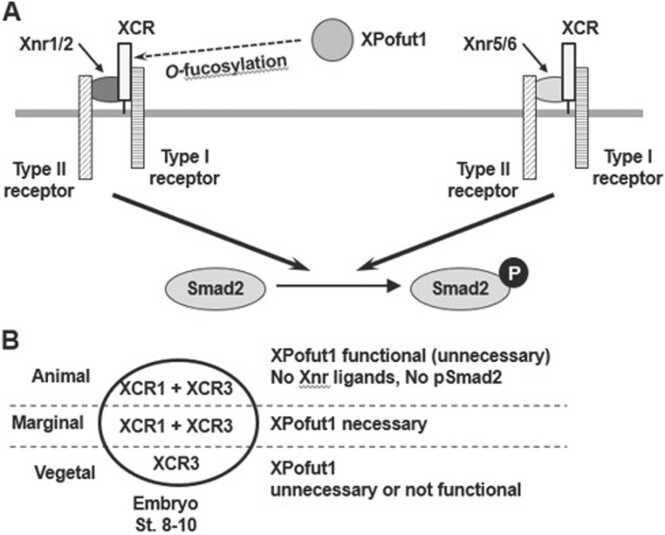
A schematic model for XPofut1 functional in Xnr signaling. (A) XPofut1 is necessary for Xnr1/2-induced Smad2 phosphorylation. XPofut1 may control Xnr1/2 signaling by *O*-fucosylation (dashed arrow) of XCRs, the *Xenopus laevis* EGF-CFC factors. XPofut1 is not necessary for Xnr5/6-induced Smad2 phosphorylation. (B) Spatial pattern of XPofut1 activity and XCR expression in stages 8 to 10 *Xenopus laevis* embryos. In the vegetal pole region (Vegetal), *XPofut1* transcripts are present but XPofut1 function is not necessary for Smad2 phosphorylation. In the marginal zone (Marginal), XPofut1 function is necessary for Smad2 phosphorylation. In the animal cap region (Animal), XPofut1 is functional as shown in animal cap assays (Fig. 6), yet there are no Xnr ligands or Smad2 phosphorylation in this region. See “Discussion” for details.

In *Xenopus laevis* embryos, Smad2 phosphorylation is initiated by Xnr5/6 and Vg1 and then increased by Xnr1/2 that are target genes of Xnr5/6 and Vg1 (Luxardi et al., 2010, Skirkanich et al., 2011, Takahashi et al., 2000). *Xnr5/6* transcripts are first expressed in the vegetal pole region of pre-MBT embryos, restricted to the vegetal pole region, and disappear before the onset of gastrulation. Once the expression of *Xnr1/2* is induced at the onset of MBT, *Xnr1/2* transcripts are distributed in the marginal zone and the vegetal pole region of blastula and early gastrula. Double knockdown of *Xnr5/6* causes defects in mesoderm formation and gastrulation movement. Double knockdown of *Xnr1/2* also causes defects in gastrulation movement, but the effects of *Xnr1/2* knockdown on mesodermal gene expression are less severe than *Xnr5/6* knockdown. These results led to a hypothesis that *Xnr5/6* are required for mesoderm induction, and *Xnr1/2* are additionally required for proper gastrulation movement (Luxardi et al., 2010). Our results suggest that *XPofut1* knockdown does not abolish Smad2 phosphorylation but it does cause reduction and aberrant temporal patterns of Smad2 phosphorylation. *XPofut1* morphants do not show pronounced gastrulation defects, but it does display axial truncation in neurula, which is an indication of reduced mesoderm formation and/or reduced gastrulation movement. Our results indicate that *XPofut1* is required in the marginal zone, where *Xnr1/2* transcripts are localized, and for Xnr1/2-induced Smad2 phosphorylation. However, *XPofut1* is not necessary in the vegetal pole region, where *Xnr5/6* transcripts are localized, and for Xnr5/6-induced Smad2 phosphorylation. Taken together, we propose that *XPofut1* controls axial patterning by regulating Xnr1 and Xnr2 signaling, but *XPofut1* is not necessary for Xnr5/6 signaling (Fig. 7A). More detailed analyses of the effects of *XPofut1* knockdown in different regions of the embryo on the spatial and temporal patterns of Smad2 phosphorylation are needed to understand the roles of *XPofut1* during embryogenesis.

### Modulation of Xnr Signaling by *XPofut1*

Pofut1 catalyzes *O*-fucosylation of conserved Ser/Thr residues (C_2_-XXXX**S/T**-C_3_) in EGF-like repeats (Ajima et al., 2017, Pandey et al., 2019). Human and mouse Cripto, which are EGF-CFC factors, have been shown to be *O*-fucosylated (Schiffer et al., 2001, Shi et al., 2007), and XCR1/2/3 all have potential *O*-fucosylation target sequence (C_2_-XNGGT-C_3_) in their EGF-like repeats (Dorey and Hill, 2006). For human and mouse Cripto, substitution of the conserved Thr residue, to which *O*-fucose is attached, to Ala renders Cripto nonfunctional for Nodal signaling (Schiffer et al., 2001). However, mouse *Pofut1* null embryos do not show typical phenotypes of *Nodal* or *Cripto* null mutant but display phenotypes consistent with deficiencies in the canonical Notch signaling (Shi et al., 2007). The results also indicate that the target Thr residue itself, but not *O*-fucose, is important for Nodal signaling in mammals (Shi et al., 2007).

In contrast to mammalian Pofut1, we found evidences that XPofut1 modulates Smad2 phosphorylation induced by a subset of Xnr ligands. Why do mammalian and *Xenopus laevis* Pofut1 show different function in the regulation of Nodal signaling? During the period of germ layer formation and gastrulation, mammalian embryos express only 1 Nodal ligand and only 1 EGF-CFC factor (Cripto), whereas *Xenopus laevis* embryos express 5 nodal-related ligands (Xnr1/2/4/5/6) and 2 EGF-CFC factors (XCR1 and XCR3) (Hill, 2022). It is intriguing to postulate that amphibian Pofut1 may have gained a function to control/distinguish signaling by multiple nodal-related ligands or that mammalian Pofut1 may have lost the ability to control signaling by single Nodal ligand. Zebrafish embryos express 2 nodal-related ligands (Cyclops and Squint) and only 1 EGF-CFC factor (Oep) during germ layer formation and gastrulation (Hill, 2022). Examining the function of Pofut1 on Nodal signaling in zebrafish and other vertebrate embryos may shed light on understanding the basis of discrepancy between the function of mammalian and *Xenopus Pofut1* in the control of Nodal signaling.

How does *XPofut1* modulate signaling for only a subset of Xnr ligands? *O*-fucosylation has been shown to modulate the ligand-binding ability of at least one receptor protein. *O*-fucosylation of Notch in *Drosophila* alters ligand-binding affinity of Notch (Pandey et al., 2019). Can *O*-fucosylation modulate Nodal binding to its receptors or coreceptors? Each Xnr ligand may have different requirements, such as different types of XCR proteins (XCR1 vs XCR3) or *O*-fucosylation of XCR proteins, for binding to their receptor/coreceptor complexes.

Our results indicate that *XPofut1* can modulate Smad2 phosphorylation with its *O*-fucosyltransferase activity. Among the components of Nodal signal transduction pathway, only EGF-CFC factors have been shown to be *O*-fucosylated, and only XCRs have *O*-fucosylation target sequences among the Xnr signaling components. Among *XCR*s, *XCR1* and *XCR3* are expressed during the period of mesoderm induction (Dorey and Hill, 2006) (Fig. 7B). We found that XPofut1 interacts with XCR proteins (Supplementary Fig. 5). Knockdown studies also show *XCR1* and *XCR3* are required for signaling by a partially overlapping subset of Xnr ligands (Dorey and Hill, 2006). Xnr1, Xnr2, and Xnr6 require both XCR1 and XCR3 to signal efficiently, while Xnr5 specifically requires XCR1 for signaling. Both *XCR1* and *XCR3* are necessary for Smad2 phosphorylation in the marginal zone, whereas only *XCR3* is necessary for Smad2 phosphorylation in the vegetal pole region. We found that *XPofut1* is required for Smad2 phosphorylation in the marginal zone, but not in the vegetal pole region (Fig. 5). Interestingly, *XPofut1* morphants display axial truncation (Fig. 3 and Supplementary Fig. 3) similar to *XCR1* morphants that show delay in blastopore lip formation (Dorey and Hill, 2006). *XCR3* morphants suffer more severe gastrulation defects. Taken together, we postulate that XPofut1 may only modulate the function of XCR1 or XPofut1 may modulate the function of XCR1 and XCR3 only in the marginal zone.

Our results suggest that *XPofut1* plays important roles in Xnr1 and Xnr2 signaling and in the marginal zone of pregastrulation embryo, where *Xnr1/2* and *XCR1/3* are required for proper development (Dorey and Hill, 2006, Luxardi et al., 2010) (Fig. 7). We also found that *XPofut1* is dispensable for Xnr5/6 signaling and in the vegetal pole region of pregastrulation embryos, where *Xnr5/6* and *XCR3* is required for proper development. Therefore, we propose that *XPofut1* regulates the signaling by a subset of Xnr ligands, Xnr1/2 but not Xnr5/6, by modulating the function of one or more XCRs through *O*-fucosylation (Fig. 7). Examining the effects of XPofut1 to the binding affinity of each Xnr ligand to its receptor/coreceptor complexes would shed light on the mechanisms of how XPofut1 regulates Xnr signaling and development.

## Author Contributions

**Yeon-Jin Kim:** Writing – original draft, Formal analysis, Data curation. **Soo Young Lee:** Writing – review & editing, supervision. **Chang-Yeol Yeo:** Writing – review & editing, Writing – original draft, Supervision. **Seung-Joo Nho:** Formal analysis, Data curation.

## Declaration of Competing Interests

The authors declare that they have no known competing financial interests or personal relationships that could have appeared to influence the work reported in this paper.

## References

[bib1] Ajima R., Suzuki E., Saga Y. (2017). Pofut1 point-mutations that disrupt O-fucosyltransferase activity destabilize the protein and abolish Notch1 signaling during mouse somitogenesis. PLoS One.

[bib2] Blanchet M.H., Good J.A.L., Mesnard D., Oorschot V., Baflast S., Minchiotti G., Klumperman J., Constam D.B. (2008). Cripto recruits Furin and PACE4 and controls Nodal trafficking during proteolytic maturation. EMBO J..

[bib3] Blanchet M.H., Good J.A.L., Oorschot V., Baflast S., Minchiotti G., Klumperman J., Constam D.B. (2008). Cripto localizes Nodal at the limiting membrane of early endosomes. Sci. Signal..

[bib4] Calvanese L., Sandomenico A., Caporale A., Foca A., Foca G., D'Auria G., Falcigno L., Ruvo M. (2015). Conformational features and binding affinities to Cripto, ALK7 and ALK4 of Nodal synthetic fragments. J. Pept. Sci..

[bib5] Carron C., Shi D.L. (2016). Specification of anteroposterior axis by combinatorial signaling during *Xenopus* development. Wiley Interdiscip. Rev. Dev. Biol..

[bib6] Dorey K., Hill C.S. (2006). A novel Cripto-related protein reveals an essential role for EGF-CFCs in Nodal signalling in *Xenopus* embryos. Dev. Biol..

[bib7] Faure S., Lee M.A., Keller T., ten Dijke P., Whitman M. (2000). Endogenous patterns of TGFbeta superfamily signaling during early *Xenopus* development. Development.

[bib8] Harland R.M. (1991). In situ hybridization: an improved whole-mount method for *Xenopus* embryos. Methods Cell. Biol..

[bib9] Hill C.S. (2018). Spatial and temporal control of NODAL signaling. Curr. Opin. Cell. Biol..

[bib10] Hill C.S. (2022). Establishment and interpretation of NODAL and BMP signaling gradients in early vertebrate development. Curr. Top. Dev. Biol..

[bib11] Jones W.D., Mullins M.C. (2022). Cell signaling pathways controlling an axis organizing center in the zebrafish. Curr. Top. Dev. Biol..

[bib12] Lee M.A., Heasman J., Whitman M. (2001). Timing of endogenous activin-like signals and regional specification of the *Xenopus* embryo. Development.

[bib13] Luxardi G., Marchal L., Thome V., Kodjabachian L. (2010). Distinct Xenopus Nodal ligands sequentially induce mesendoderm and control gastrulation movements in parallel to the Wnt/PCP pathway. Development.

[bib14] Minchiotti G., Manco G., Parisi S., Lago C.T., Rosa F., Persico M.G. (2001). Structure-function analysis of the EGF-CFC family member Cripto identifies residues essential for nodal signalling. Development.

[bib15] Onuma Y., Yeo C.Y., Whitman M. (2006). XCR2, one of three Xenopus EGF-CFC genes, has a distinct role in the regulation of left-right patterning. Development.

[bib16] Pandey A., Harvey B.M., Lopez M.F., Ito A., Haltiwanger R.S., Jafar-Nejad H. (2019). Glycosylation of specific notch EGF repeats by O-Fut1 and fringe regulates notch signaling in *Drosophila*. Cell Rep..

[bib17] Schiffer S.G., Foley S., Kaffashan A., Hronowski X., Zichittella A.E., Yeo C.Y., Miatkowski K., Adkins H.B., Damon B., Whitman M. (2001). Fucosylation of Cripto is required for its ability to facilitate nodal signaling. J. Biol. Chem..

[bib18] Shi S., Ge C., Luo Y., Hou X., Haltiwanger R.S., Stanley P. (2007). The threonine that carries fucose, but not fucose, is required for Cripto to facilitate Nodal signaling. J. Biol. Chem..

[bib19] Skirkanich J., Luxardi G., Yang J., Kodjabachian L., Klein P.S. (2011). An essential role for transcription before the MBT in *Xenopus laevis*. Dev. Biol..

[bib20] Tadjuidje E., Kofron M., Mir A., Wylie C., Heasman J., Cha S.W. (2016). Nodal signalling in Xenopus: the role of Xnr5 in left/right asymmetry and heart development. Open Biol..

[bib21] Takahashi S., Yokota C., Takano K., Tanegashima K., Onuma Y., Goto J., Asashima M. (2000). Two novel nodal-related genes initiate early inductive events in Xenopus Nieuwkoop center. Development.

[bib22] Watanabe K., Hamada S., Bianco C., Mancino M., Nagaoka T., Gonzales M., Bailly V., Strizzi L., Salomon D.S. (2007). Requirement of glycosylphosphatidylinositol anchor of Cripto-1 for trans activity as a Nodal co-receptor. J. Biol. Chem..

[bib23] Yabe S., Tanegashima K., Haramoto Y., Takahashi S., Fujii T., Kozuma S., Taketani Y., Asashima M. (2003). FRL-1, a member of the EGF-CFC family, is essential for neural differentiation in Xenopus early development. Development.

[bib24] Yeo C.Y., Chen X., Whitman M. (1999). The role of FAST-1 and Smads in transcriptional regulation by activin during early Xenopus embryogenesis. J. Biol. Chem..

[bib25] Zahn N., James-Zorn C., Ponferrada V.G., Adams D.S., Grzymkowski J., Buchholz D.R., Nascone-Yoder N.M., Horb M., Moody S.A., Vize P.D. (2022). Normal table of Xenopus development: a new graphical resource. Development.

