# Supplementary material for: Protein-O-fucosylation of coreceptors may be required for Nodal signaling in Xenopus
Source: Mol Cells. 2025 Mar 3;48(5):100207. doi: 10.1016/j.mocell.2025.100207 (PMC11964751; doi:10.1016/j.mocell.2025.100207)
Supplement: Supplementary file 1 — Supplementary material [file mmc1.docx]

**Supplementary Figures**

**Protein-*O*-fucosylation of co-receptors may be required**

**for Nodal signaling in *Xenopus***

Yeon-Jin Kim^1, 2, #^, Seung-Joo Nho^1,3, #^ Soo Young Lee^1,3,*^ and Chang-Yeol Yeo^1,*^

^1^Department of Life Science, College of Natural Sciences, Ewha Womans University, Seoul, Republic of Korea

^2^ICM, Building 102 4th Floor, 50 Yonsei-ro, Seodaemun-gu, Seoul, Republic of Korea

^3^Multitasking Macrophage Research Center, Ewha Womans University, Seoul, Republic of Korea

^#^ These authors contributed equally for this work.

^*^Correspondence: leesy@ewha.ac.kr, cyeoewha@gmail.com


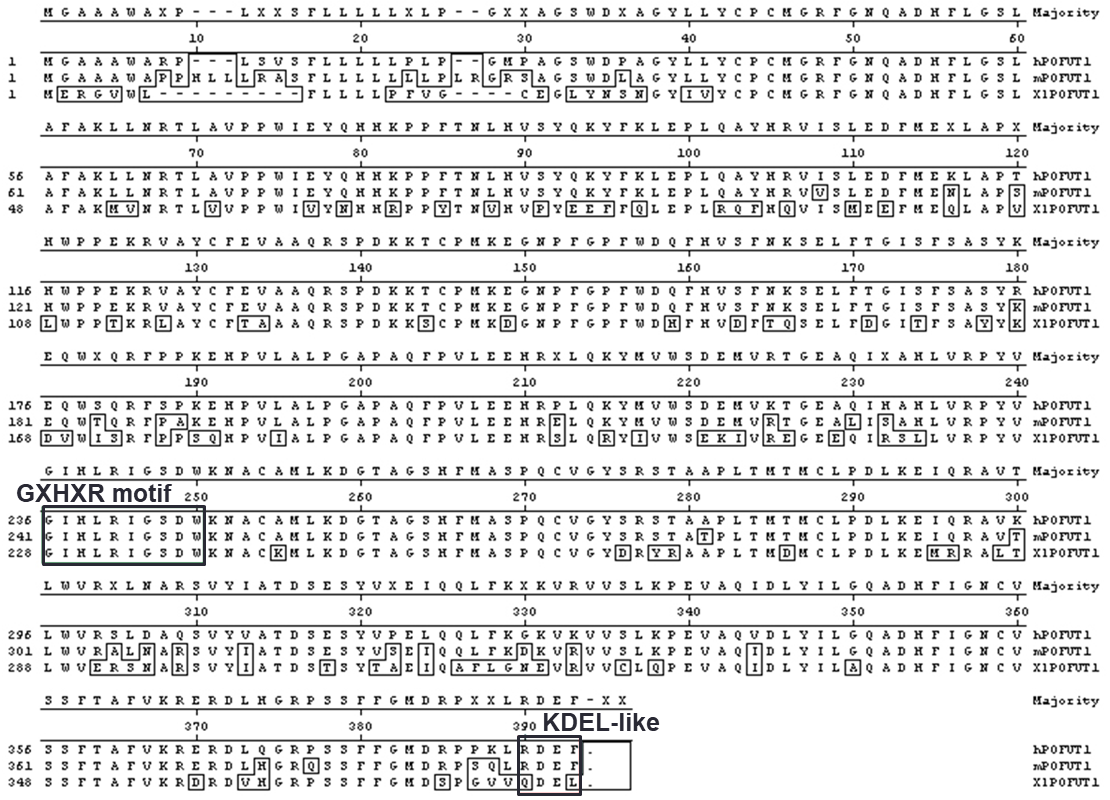


**Supplementary Figure 1. Amino acid sequence alignment of vertebrate protein-*O*-fucosyltransferase (Pofut1) proteins.**

Amino acid sequences of human POFUT1 (hPOFUT1), mouse Pofut1 (mPofut1) and *Xenopus laevis* Pofut1 (XlPofut1) proteins are aligned using the Clustal W method. Non-identical residues are marked by open boxes. GXHXR motif (a donor-substrate binding motif for glycosyltransferases) and KDEL-like ER retention sequence are indicated. Dashes denote gaps for maximum homologous alignment.


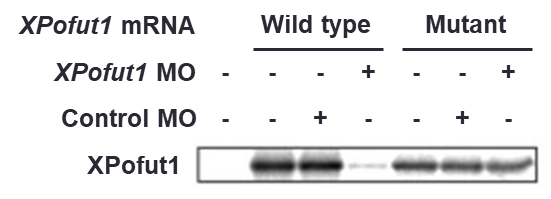


**Supplementary Figure 2. Antisense morpholino (MO) of *XPofut1* blocks the translation of *XPofut1* mRNA.**

*In vitro* translation reaction was performed with rabbit reticulocyte lysates, L-[^35^S]-Methionine, and indicated combinations of control MO (*XPofut1-sense* MO), *XPofut1* MO, wild type *XPofut1* mRNA (Wild type) containing the target sequence of *XPofut1* MO, and mutant *XPofut1* mRNA (Mutant) containing alternative codons in the *XPofut1* MO target sequence. *In vitro* translation reactions are subjected to SDS-PAGE followed by autoradiogram.


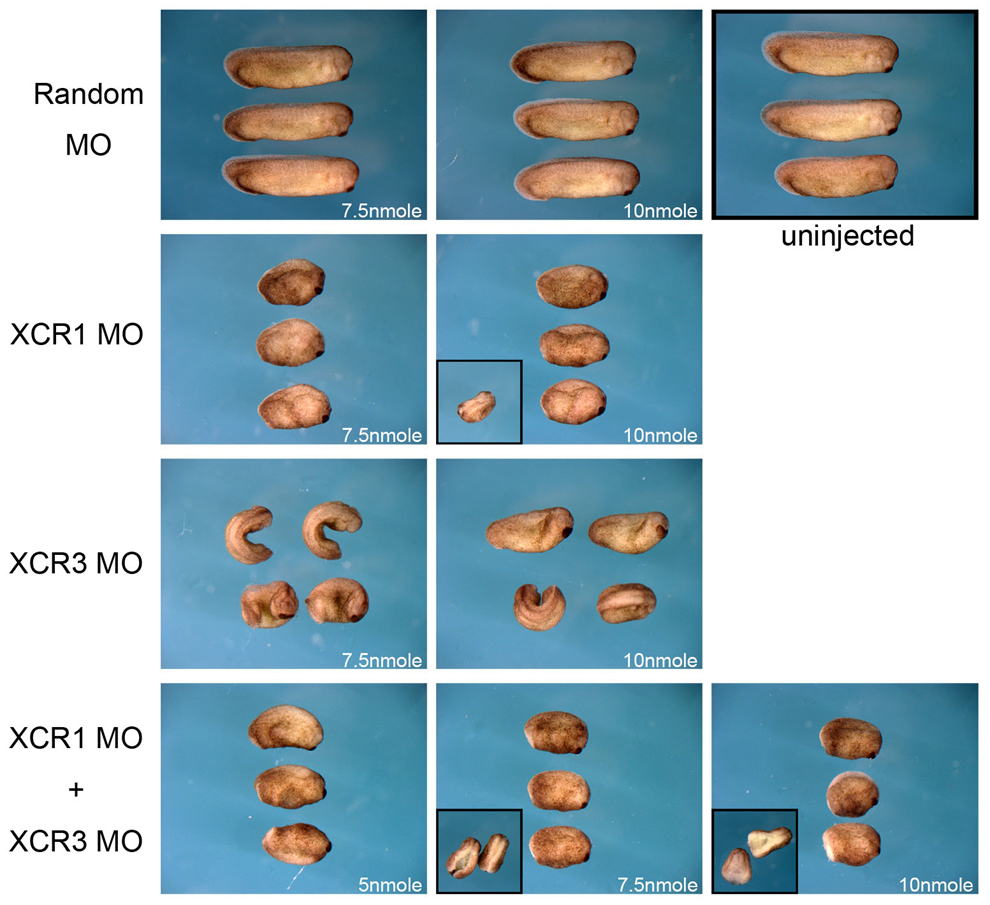


**Supplementary Figure 3. Depletion of *XCR1* and/or *XCR3* inhibits axial patterning.**

Indicated amounts and combinations of *XCR1* and *XCR3* MO (5~10 nmole total amount of MO/embryo) were injected into the marginal zone at 2-cell stage and the embryos were cultured until stage 27. Photographs in insets are dorsal views of embryos showing open neural tube.


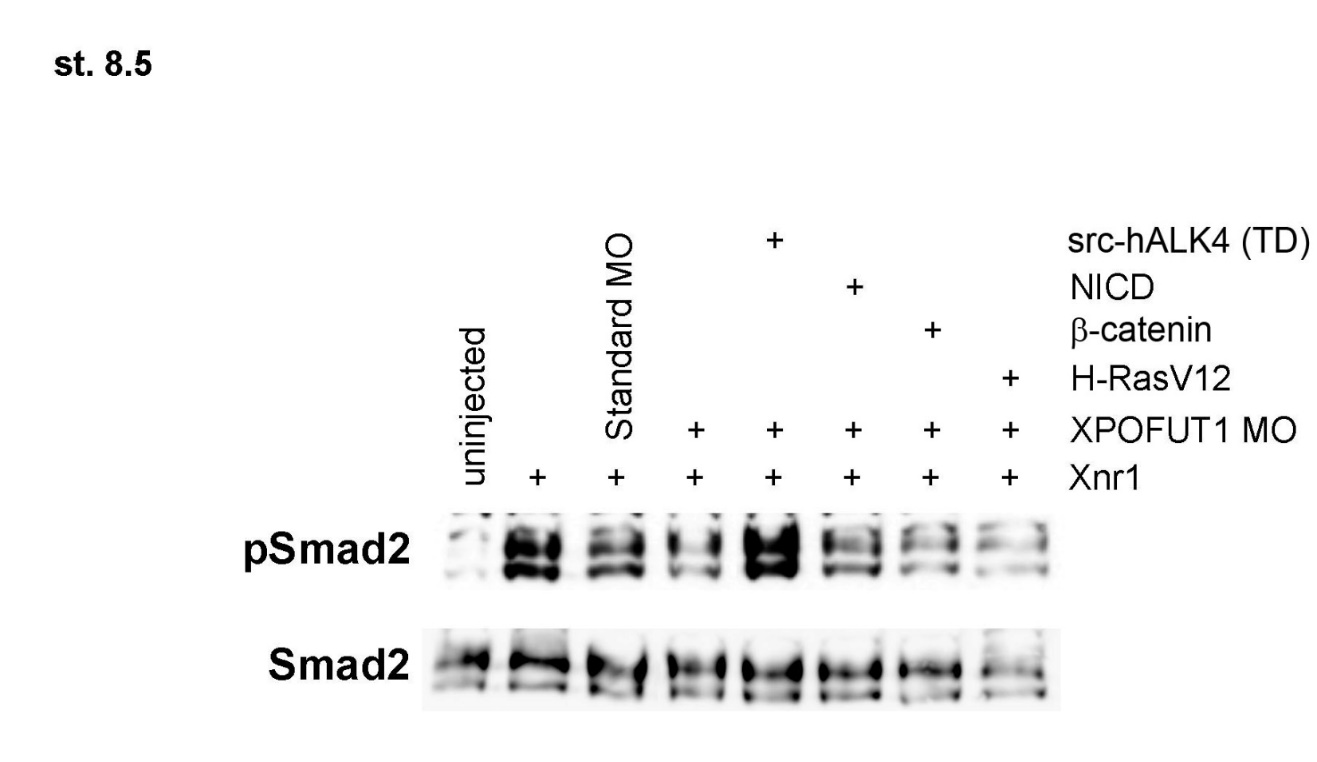


**Supplementary Figure 4. Developmentally important signaling pathways other than Nodal pathway do not relieve the inhibitory effect of *XPofut1* MO on Smad2 phosphorylation.**

mRNA (250 pg/embryo) encoding src-hALK4(TD) (a constitutively active Acvr1b fusion protein), NICD (notch intracellular domain), β-catenin, or H-RasV12 (a constitutively active Ras mutant protein) was injected into the animal region of 2-cell embryos with indicated combinations of *XPofut1* MO (5 nmole/embryo) and *Xnr1* mRNA (125 pg/embryo). Animal caps were explanted at stage 8.5, harvested at stage 9.5, and lysed. Animal cap extracts are analyzed by immunoblotting for phospho-Smad2 (pSmad2). Levels of total Smad2 are also compared.


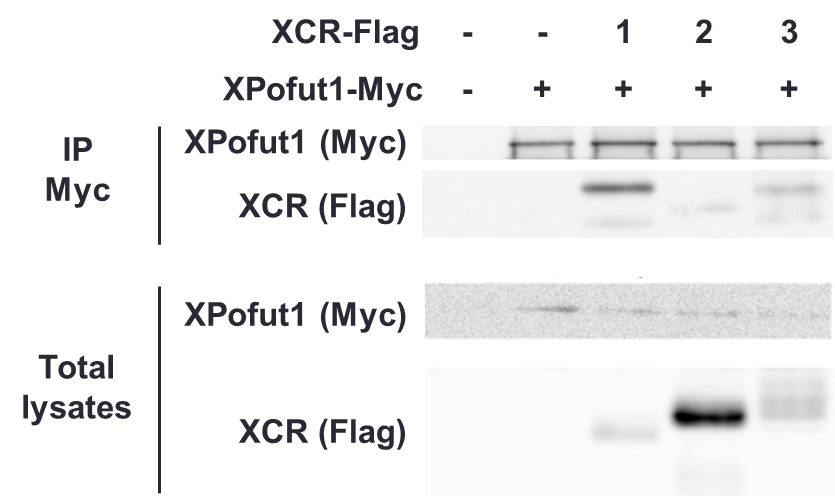


**Supplementary Figure 5. XCR proteins interact with XPofut1.**

Indicated combinations of mRNAs (250 pg each/embryo) encoding a C-terminal Myc-tagged XPofut1, and C-terminal Flag-tagged XCR 1, 2, or 3 were injected into the marginal region of 2-cell embryos and the embryos were cultured until stage 10. Embryo extracts were subjected to immunoprecipitation for XPofut1 (IP Myc) and immunoprecipitated proteins were subjected to SDS-PAGE. The interaction between XPofut1 and XCR proteins were examined by co-immunoprecipitation of XCR proteins [anti-Flag immunoblotting; IP Myc, XCR (Flag)]. Levels of XPofut1 and XCR proteins in total lysates are also examined.
